# Rice pest identification based on multi-scale double-branch GAN-ResNet

**DOI:** 10.3389/fpls.2023.1167121

**Published:** 2023-04-14

**Authors:** Kui Hu, YongMin Liu, Jiawei Nie, Xinying Zheng, Wei Zhang, Yuan Liu, TianQiang Xie

**Affiliations:** ^1^ School of Computer and Information Engineering, Central South University of Forestry and Technology, Changsha, China; ^2^ Research Center of Smart Forestry Cloud, Central South University of Forestry and Technology, Changsha, China; ^3^ School of Animal Science, South China Agricultural University, Guangzhou, China; ^4^ Business School of Hunan Normal University, Changsha, China

**Keywords:** smart agriculture, rice diseases and insect pests, deep learning, image recognition, ResNet, data enhancement

## Abstract

Rice production is crucial to the food security of all human beings, and how rice pests and diseases can be effectively prevented in and timely detected is a hotspot issue in the field of smart agriculture. Deep learning has become the preferred method for rice pest identification due to its excellent performance, especially in the aspect of autonomous learning of image features. However, in the natural environment, the dataset is too small and vulnerable to the complex background, which easily leads to problems such as overfitting, and too difficult to extract the fine features during the process of training. To solve the above problems, a Multi-Scale Dual-branch structural rice pest identification model based on a generative adversarial network and improved ResNet was proposed. Based on the ResNet model, the ConvNeXt residual block was introduced to optimize the calculation ratio of the residual blocks, and the double-branch structure was constructed to extract disease features of different sizes in the input disease images, which it adjusts the size of the convolution kernel of each branch. In the complex natural environment, data pre-processing methods such as random brightness and motion blur, and data enhancement methods such as mirroring, cropping, and scaling were used to allow the dataset of 5,932 rice disease images captured from the natural environment to be expanded to 20,000 by the dataset in this paper. The new model was trained on the new dataset to identify four common rice diseases. The experimental results showed that the recognition accuracy of the new rice pest recognition model, which was proposed for the first time, improved by 2.66% compared with the original ResNet model. Under the same experimental conditions, the new model had the best performance when compared with classical networks such as AlexNet, VGG, DenseNet, ResNet, and Transformer, and its recognition accuracy could be as high as 99.34%. The model has good generalization ability and excellent robustness, which solves the current problems in rice pest identification, such as the data set is too small and easy to lead to overfitting, and the picture background is difficult to extract disease features, and greatly improves the recognition accuracy of the model by using a multi-scale double branch structure. It provides a superior solution for crop pest and disease identification.

## 1 Introduction

The world’s total population is projected to exceed 8.5 billion by 2030. The global available arable land area has been shrinking, with the ever-changing climate and dramatic urban expansion. With crop pest and disease problems climbing as a result of climate change, food security has certainly become an urgent issue facing the world today. According to a report on the China Crop Pest and Disease Monitoring website, the vast grain-producing areas in the Yangtze River basin saw a 26.6% year-on-year increase in crop pests and disease occurrence in 2017 (Jiang et al., 2020). In 2020 alone, the cumulative area of major crop pests and diseases in China is as high as 300 million hectares (Dhaka et al., 2021), and pests and diseases not only affect grain production but may also bring economic losses (Hasan et al., 2020).

However, there are still shortcomings in the deep learning-based crop pest recognition methods. Most of the current crop pest data sets are taken under laboratory conditions. However, in practical applications, the training samples in natural environments are images with complex backgrounds, and the models are easily affected by the complex backgrounds during the training process. The deep learning models are prone to learn irrelevant features in complex backgrounds and neglect the extraction of minor diseases in training. (Fan et al., 2021). Moreover, under natural environmental conditions, the crop leaves in the field are often under strong light and swaying motion, which affects the extraction of disease features by the model (Zhang et al., 2019). At the same time, the sample size of the data set taken from the actual scene is too small, which is prone to overfitting during training and can lead to a sharp decrease in recognition accuracy during model validation.

To address the above problems, this study proposes a Multi-Scale Dual-branch structure rice pest recognition model based on Generative Adversarial Network and improved ResNet. Firstly, data pre-processing methods such as random brightness and motion blur are used to simulate the state of crop leaves in complex environments, so that the deep learning model can train such complex sample images in advance of the training process and strengthen the generalization ability and robustness. Then, data enhancement is performed on the acquired 5932 rice pest images to expand the dataset to 20,000 to alleviate the overfitting phenomenon in training. In the model construction, the input images are first enhanced with data using generative adversarial networks. The residual module in ConvNeXt was introduced into the ResNet model to optimize the calculation ratio of the residual blocks in the ResNet model to better extract the minute disease features while avoiding the occurrence of overfitting imagination and improving the recognition accuracy. Secondly, a multi-scale-two- branch structure is constructed to extract disease features at different scales using convolutional kernels at different scales, then perform feature fusion, and finally output the classification results through the Softmax layer to solve the problem of difficult extraction of tiny diseases caused by complex backgrounds. The new model is feasible and advanced in rice pest recognition and provides a reference for realizing rice pest recognition in complex environments.

The objectives of the current study are as follows.

(1) We propose a method to pre-process and enhance the training dataset. Firstly, the natural complex background is simulated to reduce the effect of strong light and wind on disease image acquisition, which enhances the flexibility of the sensor for crop disease image acquisition. Then the dataset is enhanced using image enhancement to solve the problem of too few rice disease samples, which leads to overfitting.
(2) The original ResNet model was improved, and the model recognition accuracy was improved by optimizing the residual block calculation ratio and reducing the number of computational parameters.
(3) A multi-scale dual-branch structure rice pest identification model based on generative adversarial network and improved ResNet was constructed. The number of training samples is increased by the generative adversarial network to alleviate the overfitting phenomenon, and the dual-branch structure is used to reduce the influence of the complex background of the image on the model training and improve the extraction of minor disease features.

## 2 Literature review

Research on the application of deep learning in the field of crop pest and disease identification continues to grow, and with the continuous development of deep learning. Deep learning can achieve correct identification and timely prevention of crop pests and diseases through feature extraction and classification of disease images, which can greatly save manpower and material resources and is expected to minimize economic losses (Kamilares and Francesc, 2018; Patricio and Rieder, 2018). The process of crop pest and disease identification based on deep learning includes the collection of data sets, the construction of training models, and inference validation (Zhai et al., 2021; Zhou and Wu, 2021). Lu et al. (2021) proposed a citrus yellow dragon disease recognition model based on the Mixup algorithm and convolutional neural network, and the final model achieved 94.29% recognition accuracy through data enhancement and migration learning. Huang et al. (2021) proposed a crop leaf disease recognition model in a complex environment with ResNet as the base model, combined with an inception module to extract disease features at different scales and added attention mechanisms, and the average recognition rate reached 95.62%. Luo et al. (2021) proposed a YOLOv5-C-based method for the identification of wide Buddha’s hand pests and diseases by introducing a multi-scale feature fusion module with a recognition accuracy of 93.61% in a complex background using YOLOv5s as the base model. Yu et al. (2021) proposed a knowledge graph construction method and a graph-based rice pest retrieval algorithm for rice pests and diseases, and the diagnostic algorithm achieved an 86.25% correct rate. Chen et al. (2020) used VGGNet to initialize the weights by pre-training on a large labeled dataset, ImageNet as the research object, and then performed migration learning. The initialized weights from the pre-training were transferred to the target dataset for training, and the experiments showed that the average accuracy for rice disease image recognition under complex background conditions reached 92.00%. Yuan et al. (2021) proposed a method for mushroom recognition based on migration learning combined with the ResNet-v2 network using the feature extraction capability of the Inception module as a way to improve the fine-grained feature extraction of mushroom images, and the accuracy of phenotype recognition of fine-grained mushrooms reached 93.94%. The above deep learning-based crop pest recognition method provides an important reference for current crop pest recognition research.

## 3 Materials and methods

### 3.1 Experimental data

**Figure 1:**
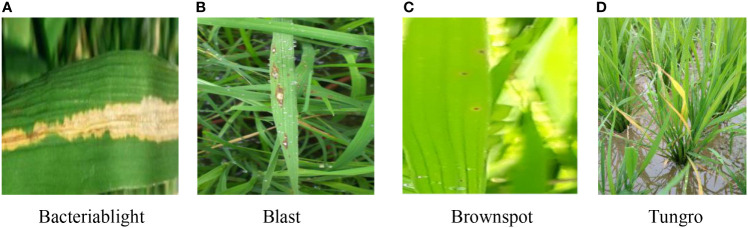
Sample images of rice diseases.

This dataset contains a total of four types of rice leaf disease images, including 1584 images of Bacteriablight, 1440 images of Blast, 1600 images of Brownspot, and 1308 images of Tungro, for a total of 5932 images, all taken under natural conditions and saved in JPG format (Sethy et al., 2020), and the images were resized to 224 × 224 pixels. The dataset was divided into training and test sets according to the ratio of 8:2, and training and test were performed under random disruption. Some of the sample images are shown in **Figure 1**.

**Figure 2:**
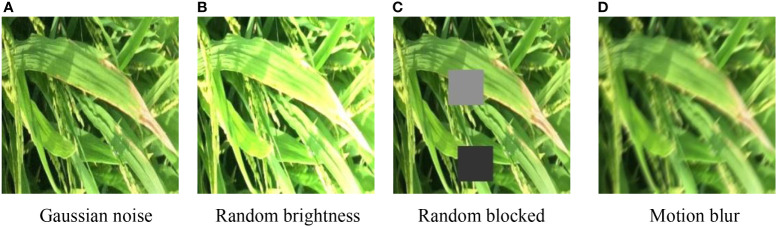
Sample images of pre-processing.

In a realistic scenario, i.e., in a rice farm, the rice leaves are often exposed to strong direct light, and the rice leaves are often interlaced and shaded by each other, and shaken by the wind. Among them, the strong direct light will affect the extraction of disease features by the model, the rice leaves are often interlaced, and the mutually blocked leaves make the disease features more difficult to extract, and the wind-blown and shaking leaves tend to make the pictures blurred. Considering the above practical factors, this paper preprocessed the original data set by image preprocessing methods such as Gaussian noise, random blocking, random brightness, and motion blur (Wu et al., 2019), so that the model learns more disease features in complex environments during the training process to achieve the purpose of simulating actual scenes and improve the accuracy of model validation. **Figure 2** shows some image preprocessing samples.

**Figure 3:**
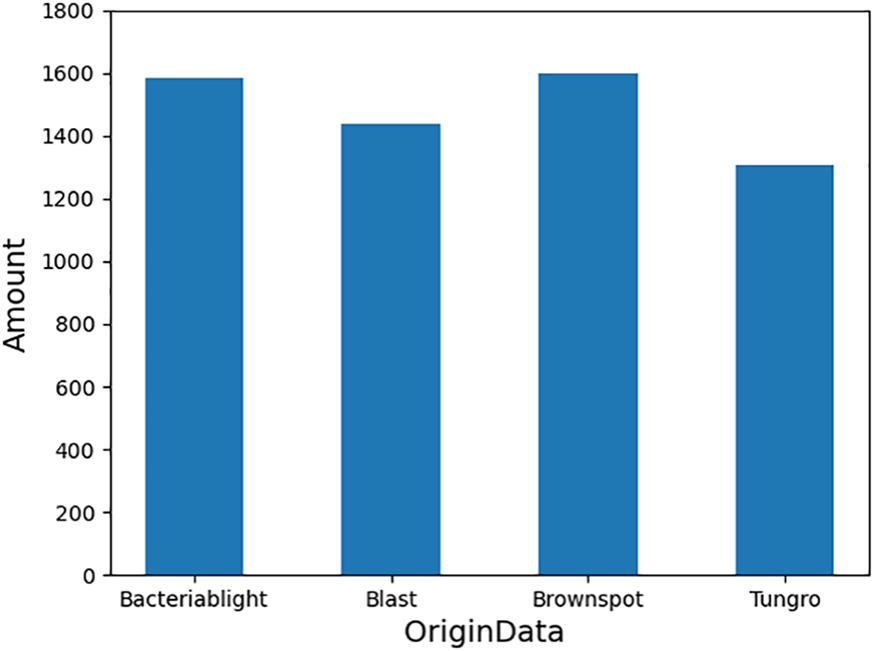
Original dataset distribution.

**Figure 4:**
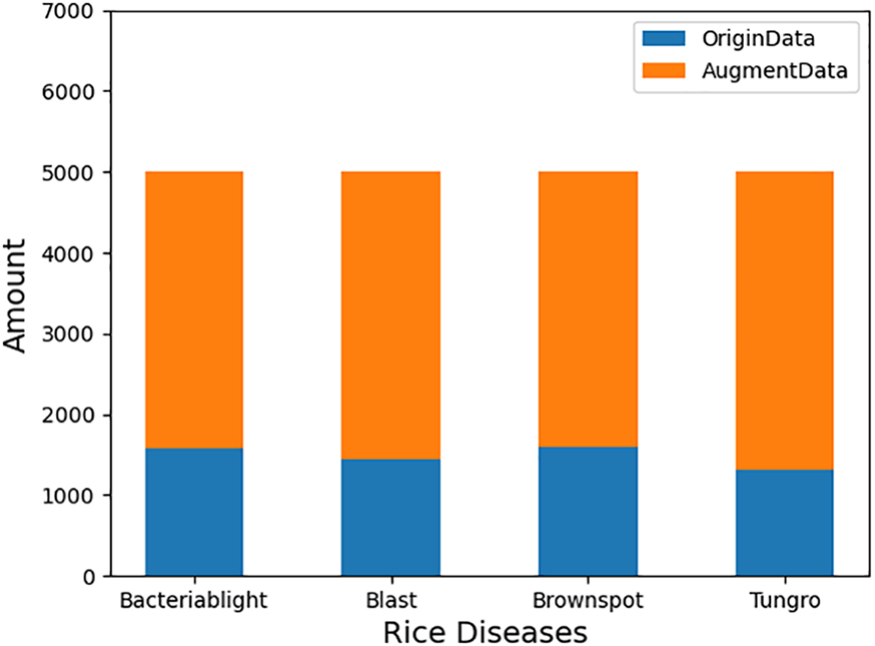
Augmented dataset distribution.

In the field of crop pest identification, data augmentation methods are mainly applied to small sample datasets or unbalanced datasets of pest and disease category image samples to increase the number of samples or to make the dataset as balanced as possible (Su et al., 2021; Liu et al., 2022). And with deep learning models becoming deeper and deeper and parameters becoming more and more massive, data augmentation methods are particularly important to enable normal training of small datasets and improve accuracy. The data enhancement methods such as mirroring, cropping, scaling, panning, and rotation (Cruz et al., 2017) are used to change the spatial location of pixels in the image and increase the number of samples to avoid overfitting without changing the content of the image through spatial geometric transformation. The rice disease dataset in this study has 4 categories of rice diseases with a total of 5932 sheets, as shown in **Table 1**. Using the data enhancement method, the samples of each disease category were expanded to 5,000 images, totaling 20,000 images, and the specific data distribution changes are shown in **Figures 3**, **4**.

**Table 1:** Rice disease dataset.

Diseases	Train	Test	Total	Aug-train	Aug-test	Aug-dataset
Bacteriablight	1265	316	1584	4000	1000	5000
Blast	1152	287	1440	4000	1000	5000
Brownspot	1280	320	1600	4000	1000	5000
Tungro	1047	261	1308	4000	1000	5000
Total	4748	1184	5932	16000	4000	20000

### 3.2 Model construction

#### 3.2.1 Convolutional neural network theory

The components of a convolutional neural network mainly include a convolutional layer, a pooling layer, and a fully connected layer. The convolutional layer computes the convolution of the input image samples through the convolutional kernel to extract the key features in the image, and the output of the convolutional layer is a representation of the input at a certain level in the spatial dimension, also called feature map (Lecun et al., 1998), and the relationship between the input and output of the convolutional layer can be expressed by equation (1).


(1) $$H_{i}=\varphi \left(H_{i-1}W_{i}+b_{i}\right)$$

where $H_{i}$ denotes the feature map of layer i, $H_{i-1}$ denotes the features of layer i-1, the features of the previous layer are input to the current convolutional layer. $W_{i}$ denotes the weight of layer i, which is the learnable parameter, is the bias of layer I and φ (•) is the activation function. The pooling layer is designed to alleviate the over-sensitivity of the convolutional layer to position and is divided into maximum pooling, average pooling and global pooling, whose output and input channels are kept consistent. The pooling layer is calculated as shown in equation (2).


(2) $$X_{j}^{l}=down\left(X_{j}^{l-1},\;s\right)$$

where $X_{j}^{l}$ represents the output features of the current pooling layer, down(•) is the downsampling function, $X_{j}^{l-1}$ is the feature vector of the previous layer, and s is the pooling window size. After the convolution operation in the convolution layer and the pooling operation in the pooling layer, the output feature vector is input into the fully connected layer to classify the extracted features. In this study, the softmax classifier is used for classification calculation, and the specific formula is shown in equation (3).


(3) $$\text{softmax}{\left(Z\right)}_{j}=e^{z_{j}}/\sum_{K=1}^{K}e^{z_{k}}\left(for\;j=1,\ldots ,\;K\right)$$

**Figure 5:**
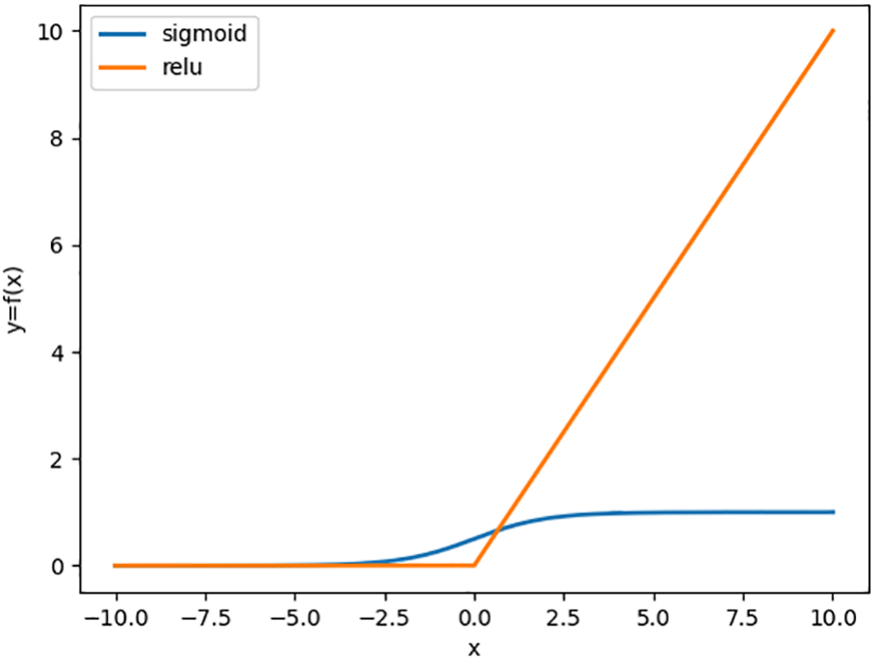
Activate function.

The softmax function is a mapping between 0 and 1. Since the sum of the output probability values of each category is not equal to 1, the e-exponential operation is utilized for the output probability of each category and then the summation is performed, which finally results in a result between 0 and 1. Since Sigmoid is extremely prone to the gradient disappearance problem, to avoid the gradient disappearance problem, the unsaturated activation function ReLU is used as the activation function in this study for this experiment (Krizhevsky et al., 2012). The function comparison curves of Sigmoid and ReLU are shown in **Figure 5**.

Considering that this study is a multiclassification experiment, stochastic gradient descent is used as the optimizer (Lu et al., 2017). The specific mathematical formulation and descent diagram are shown in equation (4).


(4) $$\theta_{j}:=\theta_{j}+\alpha \left(y^{\left(i\right)}-h_{\theta }\left(x^{\left(i\right)}\right)\right)x_{j}^{\left(i\right)}$$

Where. represents the step size, $h_{\theta }\left(x^{\left(i\right)}\right)$ represents the hypotheses function (hypotheses function), and the initial value of $\theta_{j}$ can be any value, and the parameters are updated by continuously iterating in the direction of gradient descent.

#### 3.2.2 ResNet residual theory

**Figure 6:**
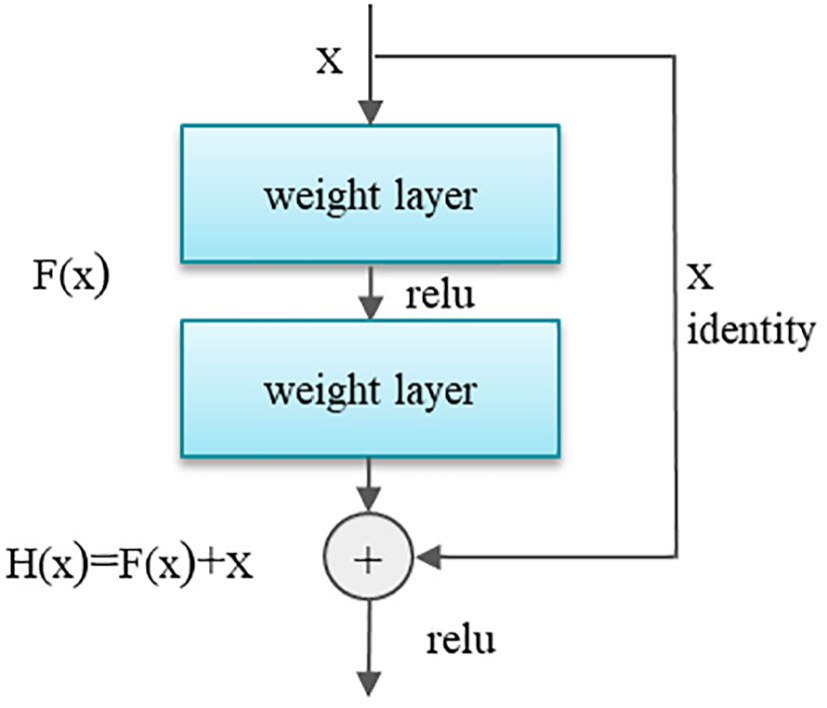
ResNet block.

In order to solve the gradient disappearance problem, He et al. (2016) proposed ResNet. Residual Network is proven to handle the vanishing gradient and effective feature learning better. This study uses the residual neural network (ResNet-50) as the base framework. ResNet-50 has 50 layers of CNNs, as well as a MaxPool and a fully connected layer with a softmax layer. resNet builds the network by stacking the remaining connections on top of each other. Even when the architecture becomes more complex, the ResNets model remains as efficient as before, making it a better choice than other architecture models(Praveen et al., 2022). The most important idea of ResNet is that the X output from the previous layer, after the convolution calculation of this layer to get the post F(X) , the X and F(X) will be added to get H(X) . The purpose of this is that when even if the. gradient tends to 0, the item X will still leave 1, cleverly avoiding the gradient during the backpropagation. The residual structure of the core in ResNet is shown in **Figure 6**.

Although the ResNet network is good for avoiding overfitting, there is still room for improvement. At the beginning of the design of the ResNet model, the model mainly consists of four Stages containing different numbers of Bottlenecks, and the ratio of the number of Bottlenecks in the Stages is largely proposed empirically, for example, the ratio of ResNet50 is 3:4:6:3, the ratio of ResNet101 is 3:4:23:3, and the ratio of ResNet152 is 3:8:36:3. It can be seen that there are also more excellent computational ratios of the number of Bottleneck in the Stage, which makes the model performance more excellent.

#### 3.2.3 ConvNeXt residuals module

**Figure 7:**
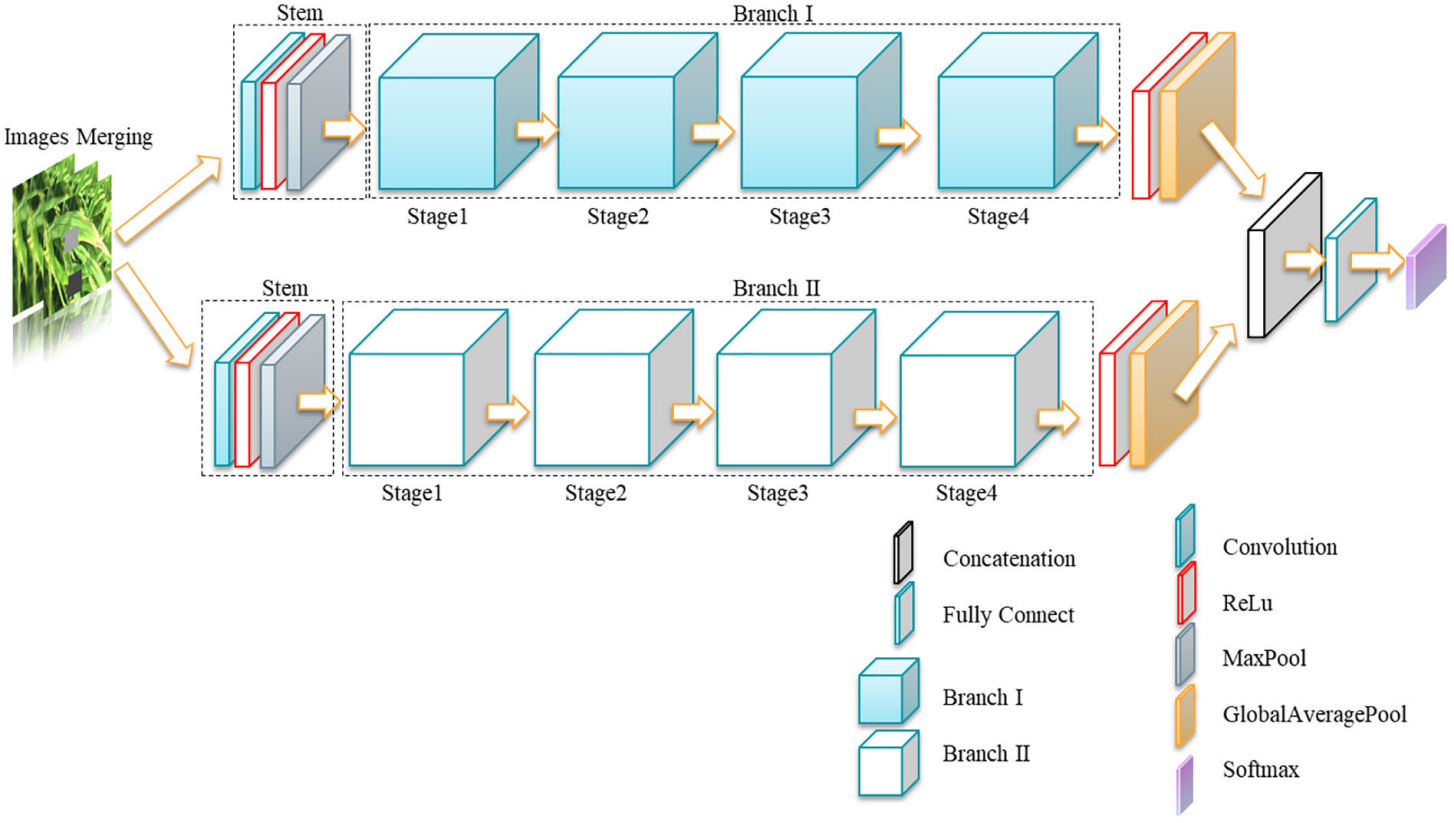
Proposed MSDB-ResNet architecture.

In 2022, Facebook AI Institute proposed the ConvNeXt convolutional neural network (Liu et al., 2022), which achieved 87.8% accuracy on the ImageNet top-1 dataset, surpassing the previous highest accuracy of 81.3% achieved by the Swin transformer (Liu et al., 2021), the ratio of residual blocks computed in the ConvNeXt network borrows the design ratio of the transformer (1:1:3:1), and the ratio of residual blocks in the ConvNext network is 3:3:9:3, which improves the accuracy of the model from 78.8% to 79.4%. The proposed residual blocks in the ConvNeXt model optimize the ratio of the number of Bottleneck in Stage. In this study, the residual blocks in ConvNext are introduced into ResNet50 as the base model, and the model accuracy of the original ResNet50 is improved on the original basis by optimizing the proportion of the number of residual blocks. The specific model parameters are shown in **Table 2**. The overall architecture of the model consists of two branches, each branch mainly consists of one stem layer and four Stages, the stem layer consists of a 7×7 convolutional layer and a 3×3 maximum pooling layer to keep the output feature resolution constant. The four Stages, i.e., res2, res3, res4, and res5 in **Table 2**, each Stage contains different numbers to the Bottleneck with a ratio of 3:3:9:3, where the specific structure of the Bottleneck is shown in **Figure 7**. This study refers to this structure as MSDB-ResNet.

**Table 2:** Model compute parameter.

	output size	MSDB-ResNet-I	MSDB-ResNet-II
stem	56×56	7×7,64,stride 2 3×3 max pool, stride 2	7×7,64,stride 2 3×3 max pool, stride 2
res2	56×56	$\begin{array}{c} 1\times 1,64\\ 3\times 3,64\\ 1\times 1,256 \end{array}\;\times 3$	
res3	28×28	$\begin{array}{c} 1\times 1,128\\ 3\times 3,128\\ 1\times 1,128 \end{array}\;\times 3$	$\begin{array}{c} 1\times 1,128\\ 5\times 5,128\\ 1\times 1,128 \end{array}\;\times 3$
res4	14×14	$\begin{array}{c} 1\times 1,256\\ 3\times 3,256\\ 1\times 1,1024 \end{array}\;\times 9$	$\begin{array}{c} 1\times 1,256\\ 5\times 5,256\\ 1\times 1,1024 \end{array}\;\times 9$
res5	7×7	$\begin{array}{c} 1\times 1,512\\ 3\times 3,512\\ 1\times 1,2048 \end{array}\;\times 3$	$\begin{array}{c} 1\times 1,512\\ 3\times 3,512\\ 1\times 1,2048 \end{array}\;\times 3$

#### 3.2.4 Multi-scale double branch structure

**Figure 8:**
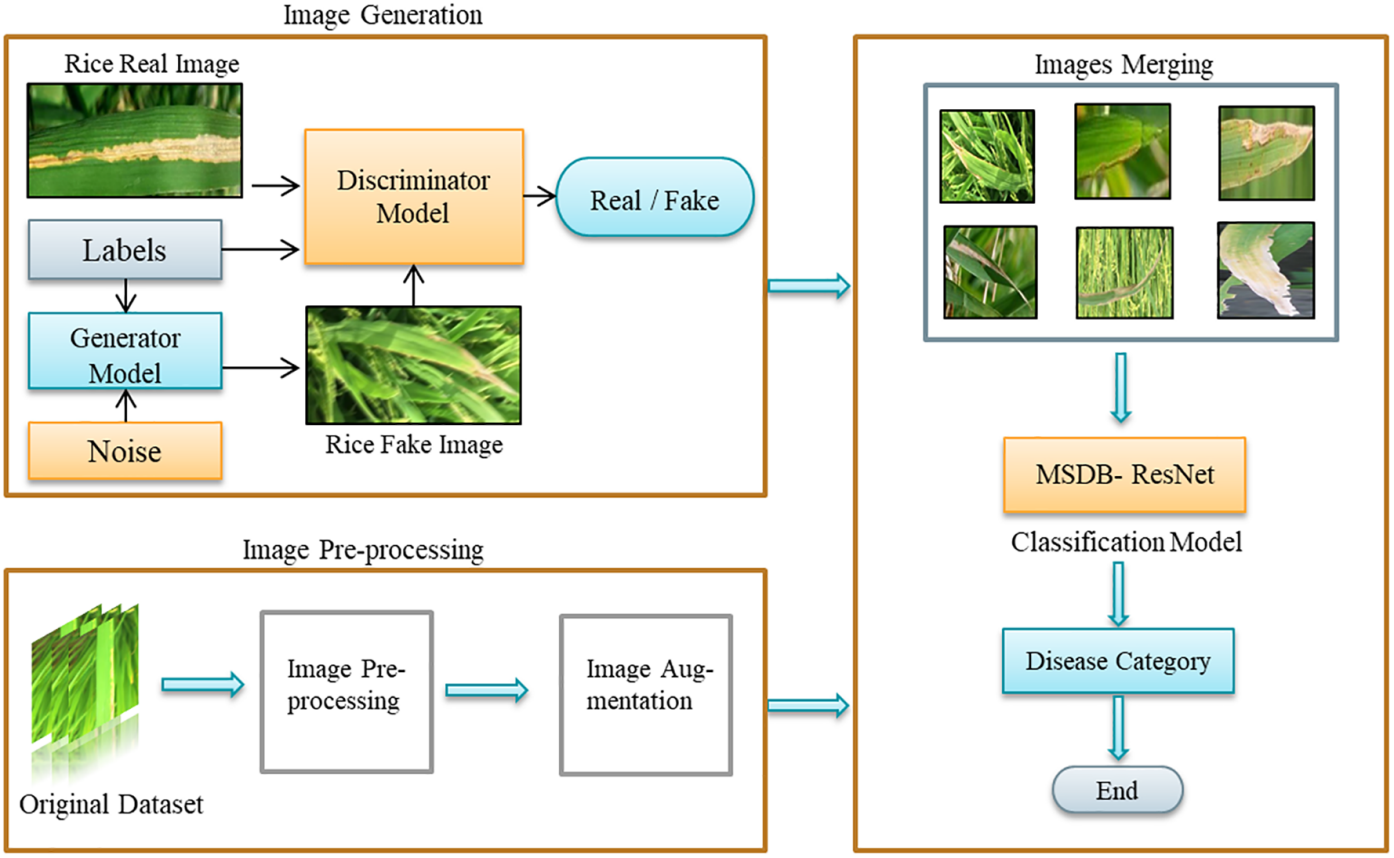
Overall process of rice disease recognition.

The overall process of rice pest identification in this study is shown in **Figure 8**. Firstly, the false rice pest images are generated using the generative adversarial network, secondly, the rice pest images preprocessed and enhanced with data from this study are fused with the false images generated by the generative adversarial network. Finally, they are input into the classification model and the classification results are output. In this study, the proposed model is referred to as GAN-MSDB-ResNet.

**Figure 9:**
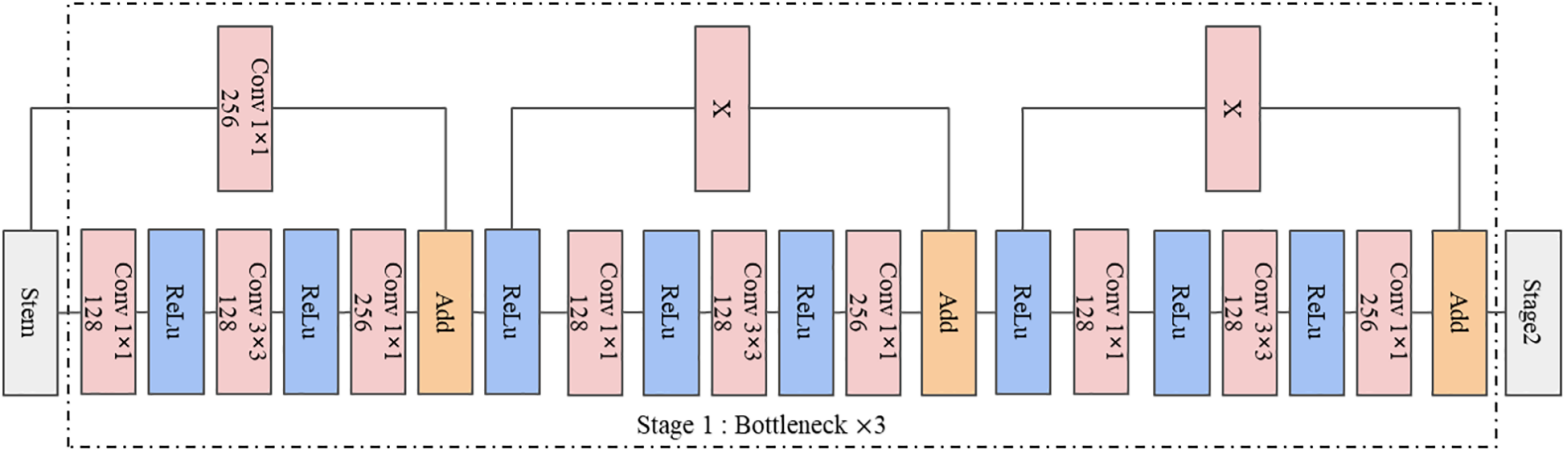
Stage of architecture.

The common sizes of convolutional kernels in the model are 1 × 1, 3 × 3, 5 × 5, and 7 × 7. Due to the different sizes of convolutional kernels, the model is prone to lose small features or easily learn the features of complex backgrounds during training, resulting in poor recognition accuracy (Guo et al., 2019). Based on this problem, this paper proposes a multi-scale dual-branch structure based on improved ResNet, with ResNet-50 as the base architecture, and constructs a dual-branch ResNet model, placing large convolutional kernels and small convolutional kernels in two different branches, respectively, to extract disease features of different sizes and reduce the influence of complex backgrounds. The model framework diagram is shown in **Figures 7**, **9**.

As shown in **Figure 7**, the input training sample images are preprocessed and enhanced, and then entered into two different branches, each of which is preceded by a stem layer consisting of a 7×7 convolutional layer, a ReLu activation layer, and a 3×3 maximum pooling layer. The structure of the Bottleneck is shown in **Figure 9**. The bottleneck consists of a residual block with a 1 × 1 convolution layer and two residual blocks with constant mapping X to avoid the occurrence of overfitting in model training. The bottleneck structure in branch 1 is composed of 1×1 and 3×3 convolutional kernels, and the bottleneck structure in branch 2 is composed of 5×5 convolutional kernels, which can extract features at different scales through different sizes of convolutional kernel operators to avoid the problem of subtle disease features being affected by complex backgrounds, resulting in key diseases not being extracted. The problem of not extracting the key disease features due to the complex background is avoided. After the input image is extracted with features by two network branches of different scales, ReLu activation and global mean pooling are performed, then it is input to concatenation layer for feature fusion, finally, it is input to the fully connected layer, and Softmax layer to output classification results.

## 4 Test results and analysis

### 4.1 Experimental environment

The experimental software environment is Windows 10 64-bit system, using Pytorch open source framework for deep learning, and Python is chosen as the programming language. The computer memory is 16 GB, equipped with AMD Ryzen 7 5800H with Radeon Graphics processor, and NVIDIA GeForce RTX 3070 Laptop graphics card to accelerate image processing.

### 4.2 Experimental parameters

In this study, the model is used SGD optimization algorithm and CrossEntropyLoss loss function. The size of the input image is 224*224, and batch size is 32, the number of training epochs is 20, the initial learning rate learning rate is set to 0.01, and the momentum is set to 0.9 and weight decay is 1e-4.

### 4.3 Model evaluation index

In this paper, the average recognition accuracy rate is used as the evaluation index of the model.


(5) $$Accuracy=\frac{1}{c}\sum_{j=1}^{c}\frac{n_{jj}}{n_{j}}\times 100\%$$

The formula c denotes the number of categories, ${\text{n}}_{\text{j}}$ denotes the number of category j, and ${\text{n}}_{\text{jj}}$ indicates the number of correct predictions in category j.

### 4.4 Analysis of data pre-processing and data enhancement test results

**Figure 10:**
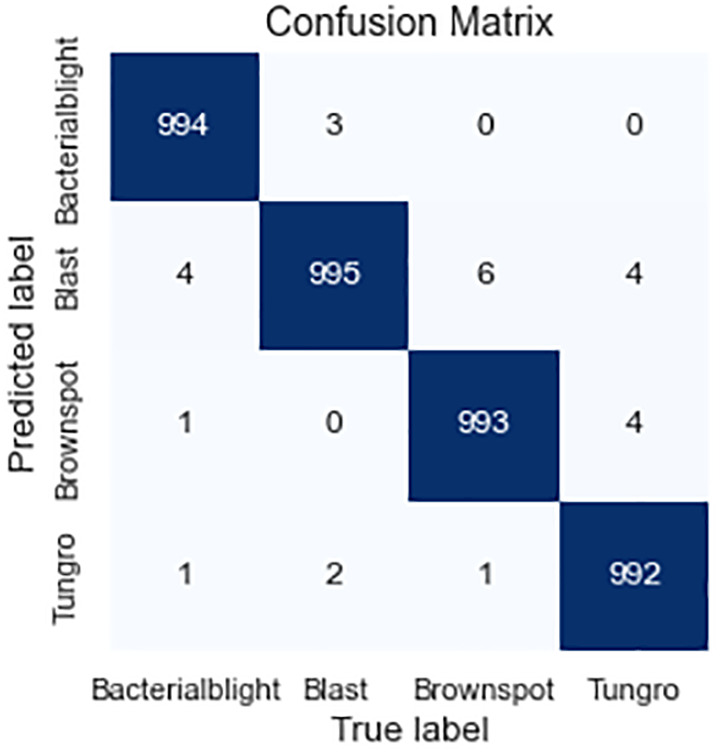
GAN-MSDB-ResNet confusion matrix.

**Figure 11:**
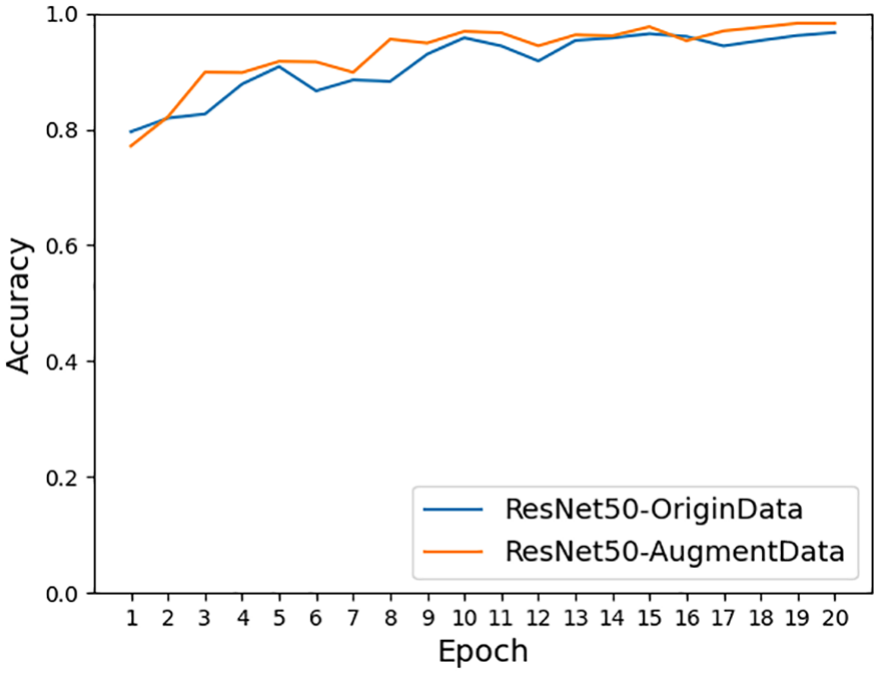
AugmentData of test comparison.

**Figure 12:**
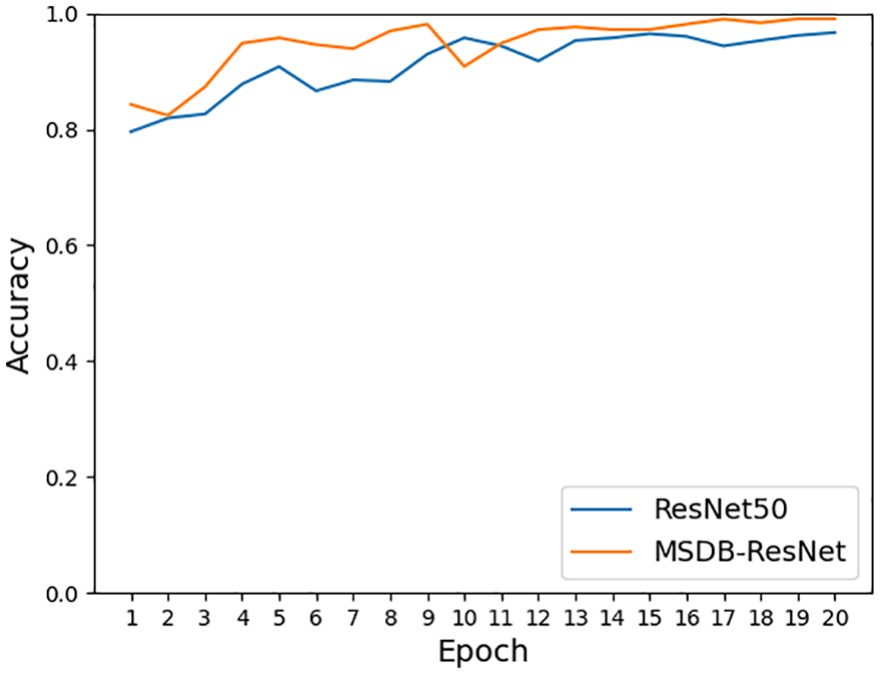
Model improvement of test comparison.

**Figure 13:**
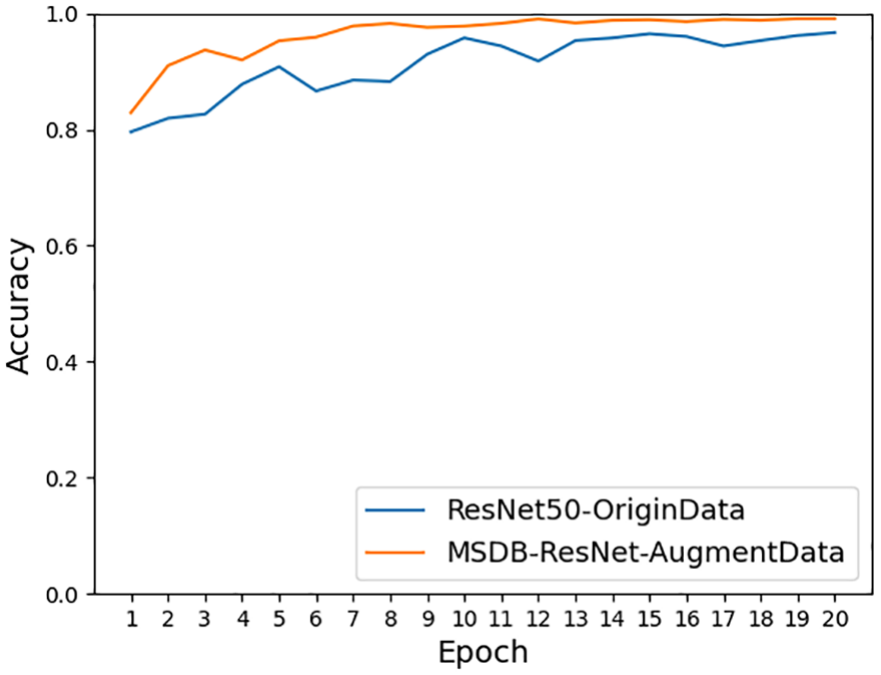
AugmentData and Model improvement.

**Figure 14:**
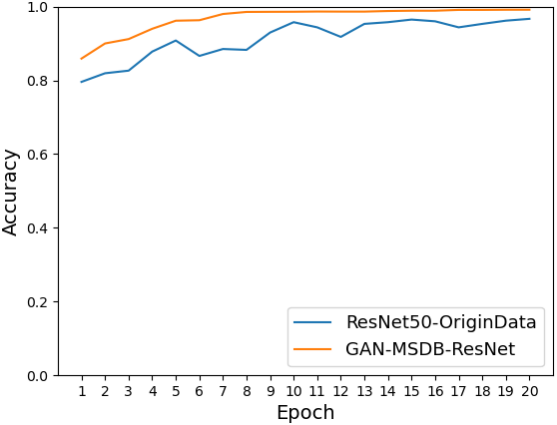
GAN and model improvement.

**Figure 15:**
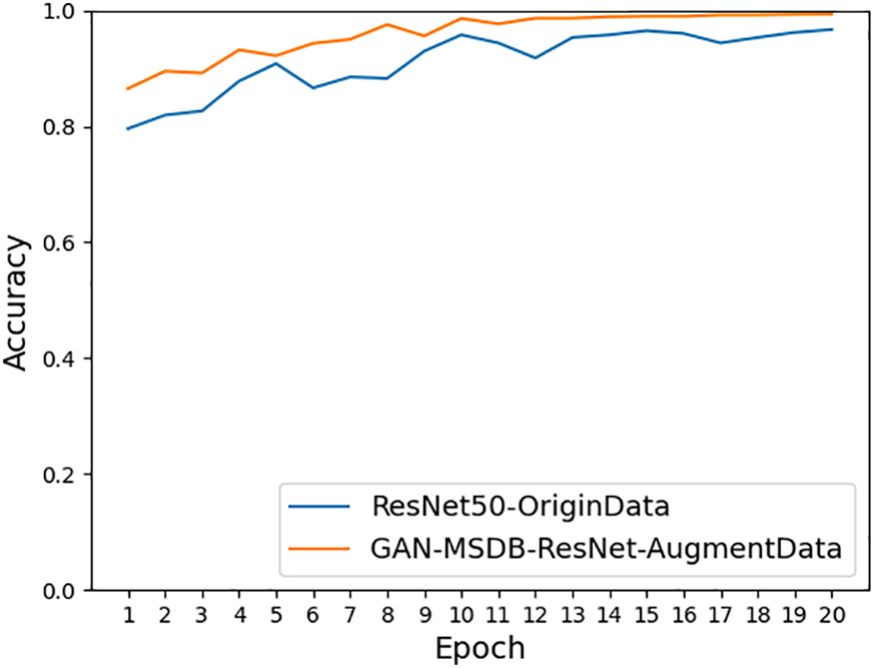
GAN AugmentData and model improvement.

**Figure 16:**
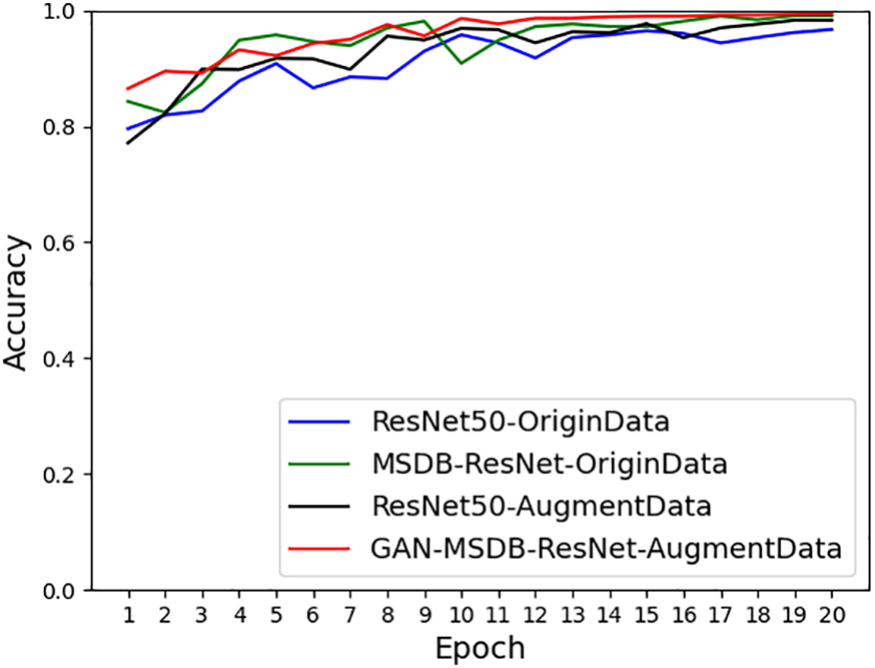
All approaches of accuracy curve.

In order to verify the performance of the model proposed in this study, several experiments were conducted on the data preprocessing and data enhancement methods, the generative adversarial network data enhancement method, and the model improvement method, respectively. Among them, the data preprocessing and data enhancement methods were compared on the base model ResNet-50 and the MSDB-ResNet model proposed in this study, respectively. The model improvement methods were tested on the original dataset and the dataset after data preprocessing and data enhancement, respectively. The generative adversarial network data enhancement method was tested on the MSDB-ResNet model. Among them, the Precision, Recall, F1-score, and Accuracy of the GAN- MSDB-ResNet model on the test dataset for four different rice pests are shown in **Table 3**, and the confusion matrix (Trevethan R et al., 2017) is shown in **Figure 10**. The results of each test and the accuracy curve comparison graphs are shown in **Table 4** and **Figures 11**–**16**.

**Table 3:** Accuracy of different approaches.

Model	Origin-Data accuracy rate/%	Data-augment accuracy rate/%
ResNet-50	96.68%	98.26%
MSDB-ResNet	99.06%	99.10%
GAN-MSDB-ResNet	99.15%	99.34%

**Table 4:** GAN-MSDB-ResNet confusion matrix.

Diseases	Precision	Recall	F1-score	Accuracy
Bacterialblight	1.00	0.99	1.00	0.994
Blast	0.99	0.99	0.99	0.995
Brownspot	0.99	0.99	0.99	0.993
Tungro	1.00	0.99	0.99	0.992

As shown in **Figure 11**, the accuracy achieved on ResNet-50 for the original dataset was 96.68%, and the accuracy achieved on ResNet-50 for the dataset after data enhancement was 98.26%, an improvement of 1.58%. It can be seen that the model accuracy can be improved by using data preprocessing methods such as Gaussian noise, motion blur, random brightness, random occlusion, and data enhancement methods. As shown in **Figure 12**, the accuracy achieved by the original dataset on ResNet-50 was 96.68%, and the accuracy achieved by introducing the ConvNet residual module, constructing the two-branch MSDB-ResNet model was 99.06%, which was a 2.38% improvement over the original ResNet-50. It can be seen that the multi-scale dual branch structure based on the improved ResNet has good performance and can significantly improve the model accuracy. As shown in **Figure 13**, the accuracy of the improved ResNet-based multiscale double branching structure (MSDB-ResNet), is 99.10% with using the data set after data enhancement, which is a significant improvement of 2.42% compared with the accuracy of 96.68% achieved by the original ResNet50 without data enhancement. To further improve the model performance, the model (GAN- MSDB-ResNet) has a model accuracy of 99.15% on the original dataset, an improvement of 2.47%, after introducing generative adversarial networks for data augmentation in the **Figure 14**. The model accuracy on the augmented dataset was 99.34%, an improvement of 2.66% in the **Figure 15**. It can be seen that the proposed Multi-Scale Dual-branch structure (GAN-MSDB-ResNet) based on a generative adversarial network and the improved ResNet has excellent performance in improving the accuracy of rice pest identification. **Figure 16** shows a composite plot of the model recognition accuracy, and the red curve shows the recognition accuracy of the improved model on the enhanced dataset.

### 4.5 Analysis of model improvement experimental results

**Figure 17:**
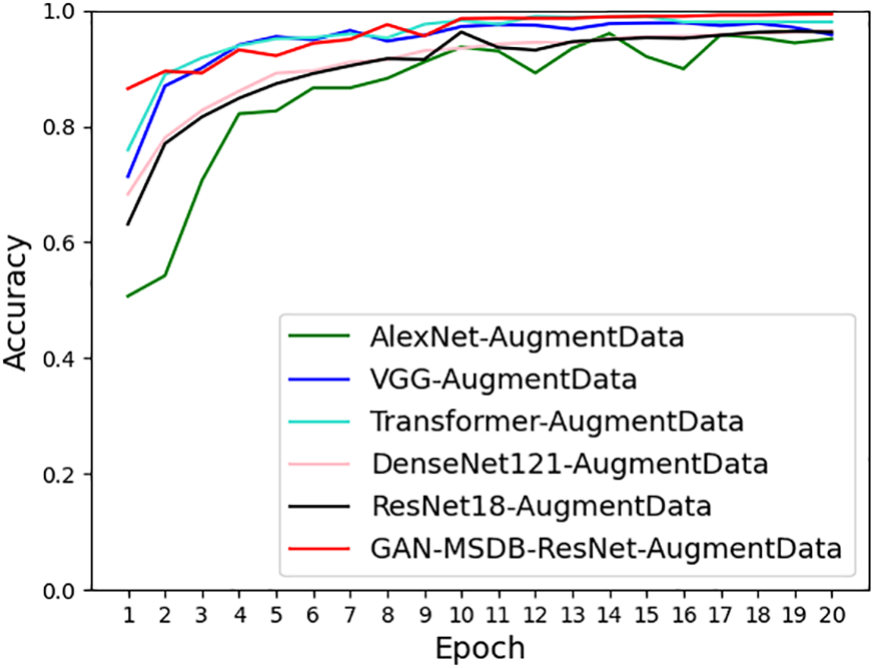
Accuracy rate curve.

**Figure 18:**
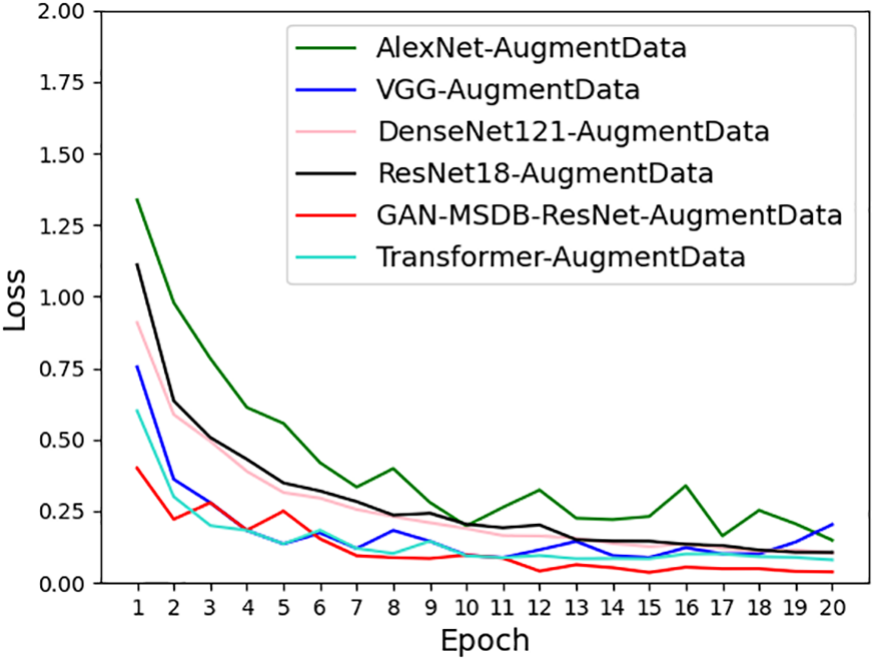
Loss curve.

To verify the robustness and generalization ability of the model, GAN-MSDB- ResNet was tested against AlexNet, VGG, DenseNet, ResNet and Transformer using the same enhanced dataset, as shown in **Table 5**, the training recognition accuracy of GAN-MSDB-ResNet was as high as 99.76%, and the test recognition accuracy was as high as 99.34%, which was the highest among highest recognition accuracy among these networks. **Figure 17** shows the comparison of accuracy curves of all models, **Figure 18** shows the comparison of loss value curves of all models. It can be seen that the improved multiscale two-branch GAN-MSDB-ResNet model has good performance for pest and disease recognition of crops in a practical environment.

**Table 5:** Accuracy and Loss of different networks.

Model	Train Loss	Test Loss	Train Acc	Test Acc
AlexNet	0.1587	0.1489	95.10%	95.07%
VGG-16	0.0379	0.2035	98.95%	95.79%
DenseNet-121	0.0691	0.1041	99.09%	96.53%
ResNet-18	0.0429	0.1064	99.16%	96.25%
Transformer	0.0291	0.0857	99.27%	97.11%
GAN-MSDB-ResNet	0.0143	0.0301	99.76%	99.34%

As shown in **Table 5**, with the same enhanced dataset, the accuracy of the validation set obtained by AlexNet is 95.07%, which is much lower than the recognition accuracy of GAN-MSDB-ResNet proposed in this study by 4.27 percentage points. The accuracy of the validation set achieved by DenseNet-121 and ResNet-18 is 96.53% and 96.25%, respectively, which is also lower than that of 99.34% achieved by GAN-MSDB-ResNet. It can be seen that the recognition accuracy achieved by DenseNet-121 and ResNet-18 containing the same residual connections on the same data set is much lower than that of GAN-MSDB-ResNet. Finally, in comparison experiments with the emerging Transformer recognition model, the accuracy of GAN-MSDB-ResNet on the validation set is higher than the accuracy of the Transformer by 2.23%. It can be seen that the model improvement method has some feasibility.

As shown in **Figures 17**, **18**, the recognition accuracy curve of GAN-MSDB-ResNet is higher than other models, and its model training convergence speed is also higher than AlexNet, VGG, DenseNet, ResNet and Transformer. The above experimental results show that the GAN-MSDB-ResNet rice pest identification model proposed in this study has good robustness and generalization ability under complex background environment.

In order to fully demonstrate the feasibility of this study, the experimental results were compared with the latest research methods in this field. As shown in **Table 6**, Xing and Lee (2020) proposed a BridgeNet-19 deep convolutional neural network for classifying 7 classes of citrus diseases with a maximum classification accuracy of 95.41%. li et al. (2020) proposed an improved GoogLeNet model to identify 10 classes of rice pests in complex backgrounds, and the optimized GoogLeNet model improved by 6.22% over the existing methods with a maximum classification accuracy of 98.91%. Krishnamoorthy and Lvnarasimha (2021) combined migration learning based on the InceptionResNetV2 model to classify three classes of rice leaf diseases with a maximum recognition accuracy of 95.67%. Chen et al. (2020) based on VGGNet, pre-trained on ImageNet after adding the Inception module. The average recognition accuracy of eight types of rice pests and diseases under complex background conditions reached 92.00%. Huang et al. (2021) proposed a MultiScale-SE-ResNet model by adding an attention mechanism based on the residual structure. The average recognition accuracy reached 95. 62% on a dataset of eight crop diseases collected in a complex field environment. By comparing with the latest research methods in this field, the GAN-MSDB-ResNet rice pest identification model proposed in this study with data enhancement through generative adversarial networks achieves 99.34% accuracy for rice pests in four types of complex backgrounds. The experimental results demonstrate the effectiveness of the method to achieve the detection of plant pests and diseases effectively.

**Table 6:** Summary comparison of crop pest and disease identification methods.

Methods	Dataset	Crop classes	Accuracy	Reference
BridgeNet-19	citrus disease 12561 images	7	95.41%	(Xing and Lee, 2020)
fine-tuned GoogLeNet	crop pest 5629 images	10	98.91%	(Li and Wang, 2020)
InceptionResNetV2	Rice disease 5200 images	4	95.67%	(Krishnamoorthy and Lvnarasimha, 2021)
VGG- transfer- Inception	Rice Maize 1000 images	8	92%	(Chen et al., 2020)
MultiScale-SE-ResNet	Rice Apple 2205 images	8	95.62%	(Huang et al., 2021)

## 5 Conclusion

The current study on deep learning-based rice pest and disease identification is critical in crop pest and disease control because they help to identify early rice diseases based on established disease datasets and improve grain yield. The proposed GAN-MSDB-ResNet rice disease recognition model has been proven a higher recognition accuracy. The study has shown that the model can learn complex disease information from images and reduce the interference of complex backgrounds, and the model recognition accuracy is greatly improved. The model has been shown to be more effective, and the model has good performance on rice pest and disease dataset, achieving 99.34% disease recognition verification accuracy on rice disease dataset with data preprocessing and data enhancement, which is 2.66% improvement compared to 96.68% recognition accuracy of ResNet-50. And it is significantly higher than the classical deep learning models such as AlexNet, VGG-16, DenseNet-121, and Transformer. Finally, the method GAN-MSDB-ResNet rice pest recognition model proposed in this study has superior performance when fully compared with the latest methods in the field of crop pest recognition. This study provides a feasible research method and an important reference for solving key problems in rice pest and disease recognition, such as complex background, too small data set, and difficult extraction of pest and disease features.

## Data availability statement

The datasets presented in this study can be found in online repositories. The names of the repository/repositories and accession number(s) can be found in the article/supplementary material.

## Author contributions

KH planned the study, provided literature reviews, prepared and conducted interviews, and drafted the manuscript. YoL and XZ reviewed, edited, conducted interviews and supervised the manuscript and lead the revision. JN and WZ reviewed and edited the manuscript and conducted interviews. YuL and TX reviewed and edited the manuscript. All authors improved the manuscript by responding to the review comments. All authors contributed to the article and approved the submitted version.

## Appendix A

Image dataset is available at: https://data.mendeley.com/datasets/fwcj7stb8r/1.

## Appendix B

Models and code are available at: https://github.com/kuihu-hk/Rice-Pest-Identification.
